# Distinct Proteomic Alterations and Solubility Profiles in Primary Tauopathies

**DOI:** 10.1002/alz70855_107594

**Published:** 2025-12-25

**Authors:** Tomas Kavanagh, Stephanie Trgovcevic, Laura Nementzik, Evgeny Kanshin, Glenda M Halliday, Beatrix Ueberheide, Eleanor Drummond

**Affiliations:** ^1^ The University of Sydney, Sydney, NSW, Australia; ^2^ NYU Grossman School of Medicine, New York, NY, USA

## Abstract

**Background:**

Primary tauopathies, including corticobasal degeneration (CBD), Pick's disease (PiD) and progressive supranuclear palsy (PSP), are characterized by tau aggregation in distinct brain regions and cell types. However, alterations of other proteins associated with these diseases remain poorly understood.

**Method:**

Sarkosyl fractionation was performed on post‐mortem frontal cortex tissue from CBD, PiD and PSP cases (*n* = 5/group) to enrich soluble and insoluble proteins. Mass spectrometry profiled the protein abundance and solubility differences. Gene set enrichment analysis and bicorrelation was used to identify dysregulated pathways and proteins associated with tau and other disease markers.

**Result:**

Tau isoforms exhibit disease specific solubility differences. CBD and PiD had high proteomic similarity, with only six proteins differing in solubility, whereas PSP was more distinct (78 differentially soluble proteins vs. CBD). Key solubility changes were observed in lysosomal regulators (e.g. LAMP2; log2FC=0.96, FDR= 0.04), postsynaptic proteins the extracellular matrix and mitochondrial proteins. S100B was elevated in the soluble fractions of CBD and PiD but not PSP (CBD vs PSP log2FC=0.68, PiD vs PSP log2FC=0.67) suggesting cellular distress was greater in the frontal cortex of these two tauopathies which correlates with higher tau pathology in these diseases. The scavenger receptor SORT1 was markedly more insoluble in CBD than either PiD or PSP (CBD vs PiD log2FC=0.60, CBD vs PSP log2FC=0.65), suggesting differences in lysosomal targeting of proteins such as progranulin. TBCC, a tubulin chaperone, was uniquely insoluble in PiD (CBD vs PiD log2FC=‐0.93, PiD vs PSP log2FC=1.02). Bicorrelation with tau revealed 791 proteins in CBD, 3,695 in PiD and 219 in PSP (ρ<‐0.7 or ρ>0.7, *p*‐value < 0.05). Notably, MOBP, a tauopathy risk gene, correlated strongly with tau in PiD (ρ = 0.82; *p*‐value = 0.004) but not in CBD or PSP.

**Conclusion:**

This study provides the first comparative analysis of protein solubility across tauopathies, revealing disease‐specific proteomic signatures and divergent mechanisms. Our findings reveal key proteins and pathways linked to tau pathology, offering insights into tauopathy pathogenesis and discriminatory biomarkers.

